# Genome-wide identification and characterization of the *TIFY* gene family in kiwifruit

**DOI:** 10.1186/s12864-022-08398-8

**Published:** 2022-03-05

**Authors:** Junjie Tao, Huimin Jia, Mengting Wu, Wenqi Zhong, Dongfeng Jia, Zupeng Wang, Chunhui Huang

**Affiliations:** 1 College of Agronomy, Jiangxi Agricultural University, Nanchang, 330045 China; 2 Wuhan Botanical Garden, Chinese Academy of Sciences, Wuhan, 430074 China

**Keywords:** Kiwifruit, TIFY, JAZ, Gene family, Psa

## Abstract

**Background:**

The *TIFY* gene family is a group of plant-specific transcription factors involved in regulation of plant growth and development and a variety of stress responses. However, the *TIFY* family has not yet been well characterized in kiwifruit, a popular fruit with important nutritional and economic value.

**Results:**

A total of 27 and 21 *TIFY* genes were identified in the genomes of *Actinidia eriantha* and *A. chinensis*, respectively. Phylogenetic analyses showed that kiwifruit *TIFY* genes could be classified into four major groups, JAZ, ZML, TIFY and PPD, and the JAZ group could be further clustered into six subgroups (JAZ I to JAZ VI). Members within the same group or subgroup have similar exon-intron structures and conserved motif compositions. The kiwifruit *TIFY* genes are unevenly distributed on the chromosomes, and the segmental duplication events played a vital role in the expansion of the *TIFY* genes in kiwifruit. Syntenic analyses of *TIFY* genes between kiwifruit and other five plant species (including *Arabidopsis thaliana*, *Camellia sinensis*, *Oryza sativa*, *Solanum lycopersicum* and *Vitis vinifera*) and between the two kiwifruit species provided valuable clues for understanding the potential evolution of the kiwifruit *TIFY* family. Molecular evolutionary analysis showed that the evolution of kiwifruit *TIFY* genes was primarily constrained by intense purifying selection. Promoter cis-element analysis showed that most kiwifruit *TIFY* genes possess multiple cis-elements related to stress-response, phytohormone signal transduction and plant growth and development. The expression pattern analyses indicated that *TIFY* genes might play a role in different kiwifruit tissues, including fruit at specific development stages. In addition, several *TIFY* genes with high expression levels during Psa (*Pseudomonas syringae* pv. *actinidiae*) infection were identified, suggesting a role in the process of Pas infection.

**Conclusions:**

In this study, the kiwifruit *TIFY* genes were identified from two assembled kiwifruit genomes. In addition, their basic physiochemical properties, chromosomal localization, phylogeny, gene structures and conserved motifs, synteny analyses, promoter cis-elements and expression patters were systematically examined. The results laid a foundation for further understanding the function of *TIFY* genes in kiwifruit, and provided a new potential approach for the prevention and treatment of Psa infection.

**Supplementary Information:**

The online version contains supplementary material available at 10.1186/s12864-022-08398-8.

## Background

The *TIFY* family is a plant-specific gene family coding for transcription factors. The *AT4G24470* gene in *Arabidopsis thaliana* was the first member of the *TIFY* gene family to be characterized and was previously known as *ZIM* (Zinc-finger protein expressed in Inflorescence Meristem) because it contains a C2C2-GATA zinc-finger structure [1, 2]. Due to presence of the highly conserved TIF[F/Y]XG (X represents any amino acid) motif in the ZIM domain-containing protein sequences, the *ZIM* family was later renamed and catalogued as the *TIFY* family [1]. The *TIFY* gene family could be further classified into four major subfamilies based on the different domain architectures and phylogenetic analyses, including TIFY, JAZ (jasmonate-ZIM-domain), PPD (PEAPOD) and ZML (ZIM/ZIM-like) [3]. The TIFY subfamily proteins contain a conserved TIFY domain, which is also shared by the other three subfamilies. In addition to the TIFY domain, the other three subfamilies contain specific and conserved domains. For example, the JAZ subfamily contains another conserved JA-associated (Jas, also named CCT_2) functional domain with a special consensus sequence SLX2FX2KRX2RX5PY near the C-terminus [4, 5]. The PPD subfamily contains a unique PPD domain in the N-terminus and a diverged Jas domain that lacks the conserved P and Y amino acids at the C-terminus region [4]. The ZML subfamily proteins contain a C-terminus C2C2-GATA zinc-finger DNA-binding domain and a CCT (CONSTANS, CO-like and TOC1) domain involved in protein-protein interaction [4].

The *TIFY* gene family plays a critical role in plant growth and development as well as in stress responses. The *TIFY* family genes are widely involved in regulating the development processes of plant organs and tissues such as stem, leaf and flower. For instance, studies have shown that *AtTIFY1* (ZIM), the first characterized member of the *TIFY* family in *Arabidopsis thaliana*, was not only related to the development of inflorescence and flowering, but also promoted the petioles and hypocotyl extension by mediating cell elongation [2, 6]. Moreover, *AtTIFY4a* (PPD1) and *AtTIFY4b* (PPD2) enhanced leaf growth by regulating lamina size and limiting curvature of the leaf blade [7], and the *AtTIFY4b* (PPD2) gene was also involved in regulating leaf flatness and lateral organ development [8, 9]. In rice, overexpression of *OsTIFY11b/OsJAZ10* could increase grain-size by enhancing accumulation and translocation of carbohydrates in the stems and leaf sheaths [10]. Another rice *JAZ* gene, *OsTIFY3/OsJAZ1*, was involved in regulation of spikelet development [11]. *Arabidopsis thaliana* with overexpression of a Jas-domain deletion version of *AtJAZ1* (*AtJAZ1*Δ*Jas*) displayed an early flowering phenotype under short day conditions, while overexpression of the *CmJAZ1-like* gene resulted in a late flowering phenotype in *Chrysanthemum morifolium* [12, 13]. Tomato plants overexpressing *SlJAZ2* exhibited accelerated vegetative growth and early flowering [14].

Apart from the critical functions in plant growth and development, the *TIFY* family genes also play important regulatory roles in defense to various abiotic and biotic stresses. In terms of abiotic stresses, the *TIFY* gene family participates in response to drought stress, salt stress, alkaline stress and other abiotic stresses. For instance, in *Arabidopsis thaliana*, overexpression of *AtJAZ7* conferred drought tolerance by regulating plant photosynthesis, redox, amino acids, phytohormones and defense metabolites [15]. In addition, OsJAZ1 was also involved in drought tolerance in rice by interacting with OsbHLH148 in the jasmonate signaling pathway [16]. In rice, *OsTIFY11a*-overexpressing plants could significantly improve tolerance to salt and dehydration stresses [17]. Overexpression of *GsJAZ2* was demonstrated to improve tolerance to alkaline stress in soybean [18]. Under high salinity conditions, the germination and growth rate of *Arabidopsis* seedlings overexpressing wheat *TdTIFY11a* were higher than wild type, showing higher salt stress tolerance [19]. In terms of biotic stresses, JAZ proteins in *Arabidopsis* played a role in response to wounding and herbivory [20]. Furthermore, transgenic bread wheat lines over-expressing *TaJAZ1* improved the resistance to powdery mildew by promoting the accumulation of reactive oxygen species [21]. In addition, the *TIFY* gene family has several other functions. For example, *AsJAZ1* in *Astragalus sinicus* was involved in nodule development and nitrogen fixation [22], and *AhJAZ1* and *AhTIFY8* in *Arachis hypogaea* also participated in the root nodule symbiosis process [23]. The multiple regulatory effects of the *TIFY* gene family in plant growth and stress resistance indicate that this family contains a large number of valuable gene resources related to plant life activities and stress resistance responses, and thus it is of great significance to mine, identify and characterize these genes.

**Fig. 1:**
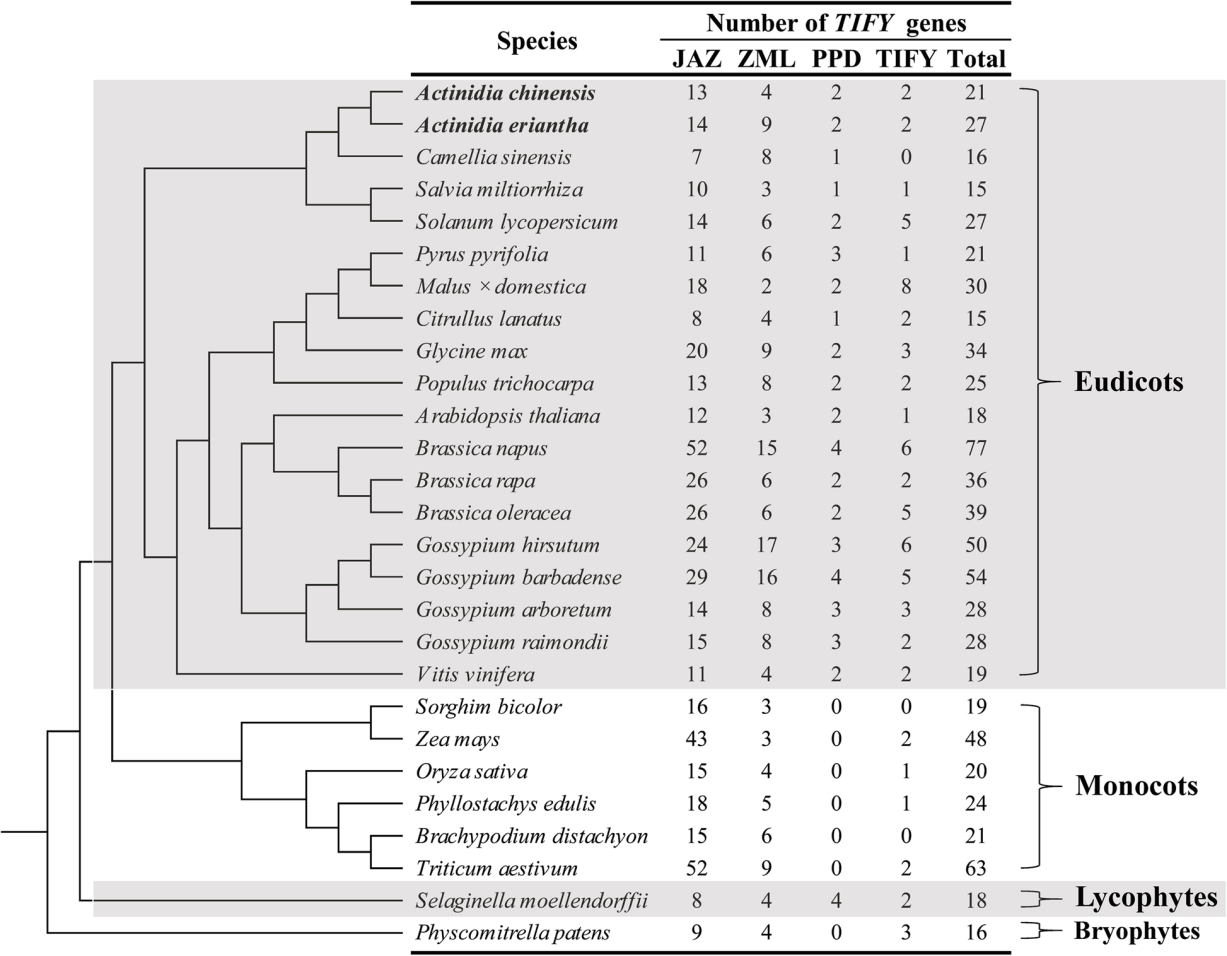
The number variations and compositions of *TIFY* gene families in different plant genomes. The plant species evolutionary relationships were redrawn according to APG (Angiosperm Phylogeny Group) IV

In recent years, a large number of plant genomes have been sequenced and released, which laid the foundation for the identification and characterization of *TIFY* gene family at the whole genome level. The *TIFY* family genes have been identified and characterized in at least 25 plant species, including *Arabidopsis* [1], *Oryza sativa* (rice) [17], *Zea mays* (maize) [24, 25], *Tricicum aestivum* (wheat) [19, 26, 27], *Vitis vinifera* (grape) [28], *Malus* × *domestica* (apple) [29], *Solanum lycopersicum* (tomato) [25, 30], *Pyrus pyrifolia* (sand pear) [31], *Citrullus lanatus* (watermelon) [32], and *Camellia sinensis* (tea) [33] (Fig. 1). A variable number of *TIFY* genes content were identified in these plant species, of which up to 77 *TIFY* genes identified in *Brassica napus* [34], while only 15 *TIFY* genes identified in *Salvia militiorrhiza* [35] and *Citrullus lanatus* [32] (Fig. 1). Most of the identified *TIFYs* in these species, especially those in eudicots, could be clustered into four subfamilies (TIYF, JAZ, ZML, PPD). The *TIFYs* in monocots plants seemed to be only grouped into three subfamilies, and no PPD subfamily members were detected in monocot species (Fig. 1). With the identification of more *TIFY* genes in plants, the understanding of *TIFY* genes is gradually deepened, and the biological functions of *TIFY* genes are gradually clarified. However, the evolution and functional divergence of the kiwifruit *TIFY* genes remained unclear until now.

Kiwifruit is a popular fresh fruit consumed worldwide, with important nutritional and economic value. Because the fruits are remarkably rich in vitamin C, dietary fiber, mineral elements and other nutrients, kiwifruit is also well known as ‘the king of fruits’. The *TIFY* gene family has a variety of important biological functions and plays a very important regulatory role in plant growth and development and stress resistance. Therefore, it is of great significance to identify and clarify the biological functions of *TIFY* gene family in kiwifruit. Currently, several kiwifruit genomes have been released, such as *Actinidia chinensis* ‘Hongyang’ [36, 37], *A. chinensis* ‘red5’ [38] and *A. eriantha* ‘White’ [39], which will be helpful in identifying and characterizing kiwifruit *TIFY* gene family.

In this study, genome-wide identification and investigation of the *TIFY* family genes were carried out from two assembled kiwifruit genomes, including *A. chinensis* ‘Hongyang’ genomes (v3.0) and *A. eriantha* ‘White’ genome. Furthermore, gene structures, conserved domains, phylogenetic analysis, chromosomal locations, cis-regulatory elements, gene synteny analyses and expression characteristics were subsequently analyzed. The results of this study will pave a way to further understanding the evolution and biological functions of kiwifruit *TIFY* genes.

## Results

### Identification of *TIFY* genes in kiwifruit

To identify all the putative *TIFY* genes in kiwifruit, the seed profile of the TIFY domain (PF06200) was used to search against the annotated proteins of *A. eriantha*, and *A. chinensis*. All the putative members were verified for the presence of the conserved TIFY domain, and finally, a total of 27 and 21 *TIFY* genes were identified in the genome of *A.eriantha* and *A. chinensis*, respectively (Fig. 1). The CDS (coding sequence) and amino acid lengths of these identified TIFY sequences varied extensively. For example, the amino acid sequence lengths of AeTIFYs and AcTIFYs varied from 112 aa (AePPD1) to 718 aa (AeJAZ13) and from 107 aa (AcJAZ13) to 764 aa (AcZML1), respectively (Additional file 1). The predicted molecular weight of AeTIFYs and AcTIFYs also varied greatly, and ranged from 12.3 kDa (AePPD1) to 81.2 kDa (AeJAZ13) and from 12.2 kDa (AcJAZ13) to 84.1 kDa (AcZML1), respectively (Additional file 1). The theoretical pI values of 19 out of 27 TIFYs in *A. eriantha* and 18 out of 21 TIFYs in *A. chinensis* were higher than 7.0, indicating that most of the kiwifruit TIFYs were alkaline proteins. Subcellular localization analysis showed that, except for the AcZML1 in *A. chinensis* predicted to be located in the mitochondrion, all other kiwifruit TIFYs were predicted to be located in the nucleus (Additional file 1). The identified kiwifruit *TIFY* genes were named on the basis of their chromosomal positions and the phylogenetic relationships with the *TIFY* genes in *Arabidopsis* and rice described below. The detail information of these kiwifruit *TIFY* family genes is available in Additional file 1.

### Phylogenetic analyses and classification of the kiwifruit *TIFY* gene family

**Fig. 2:**
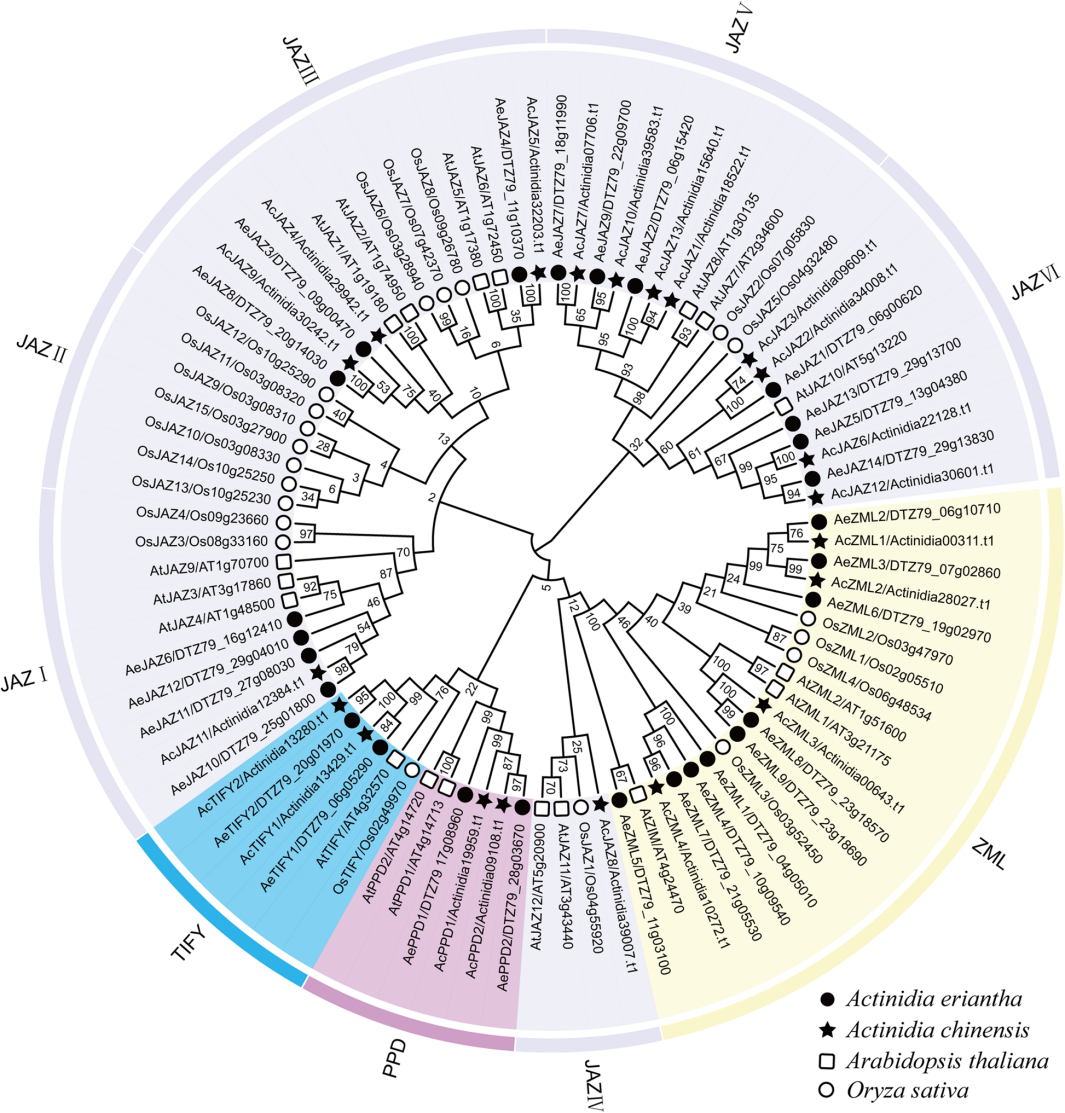
Neighbor-joining phylogenetic relationships of the TIFY proteins from kiwifruit, *Arabidopsis* and *Oryza sativa*. The phylogenetic tree was generated using MEGA-X software and the bootstrap method (1000 replicates) with full-length amino acid sequences from *A. chinensis*, *A. eriantha*, *Arabidopsis* and *O. sativa*

**Fig. 3:**
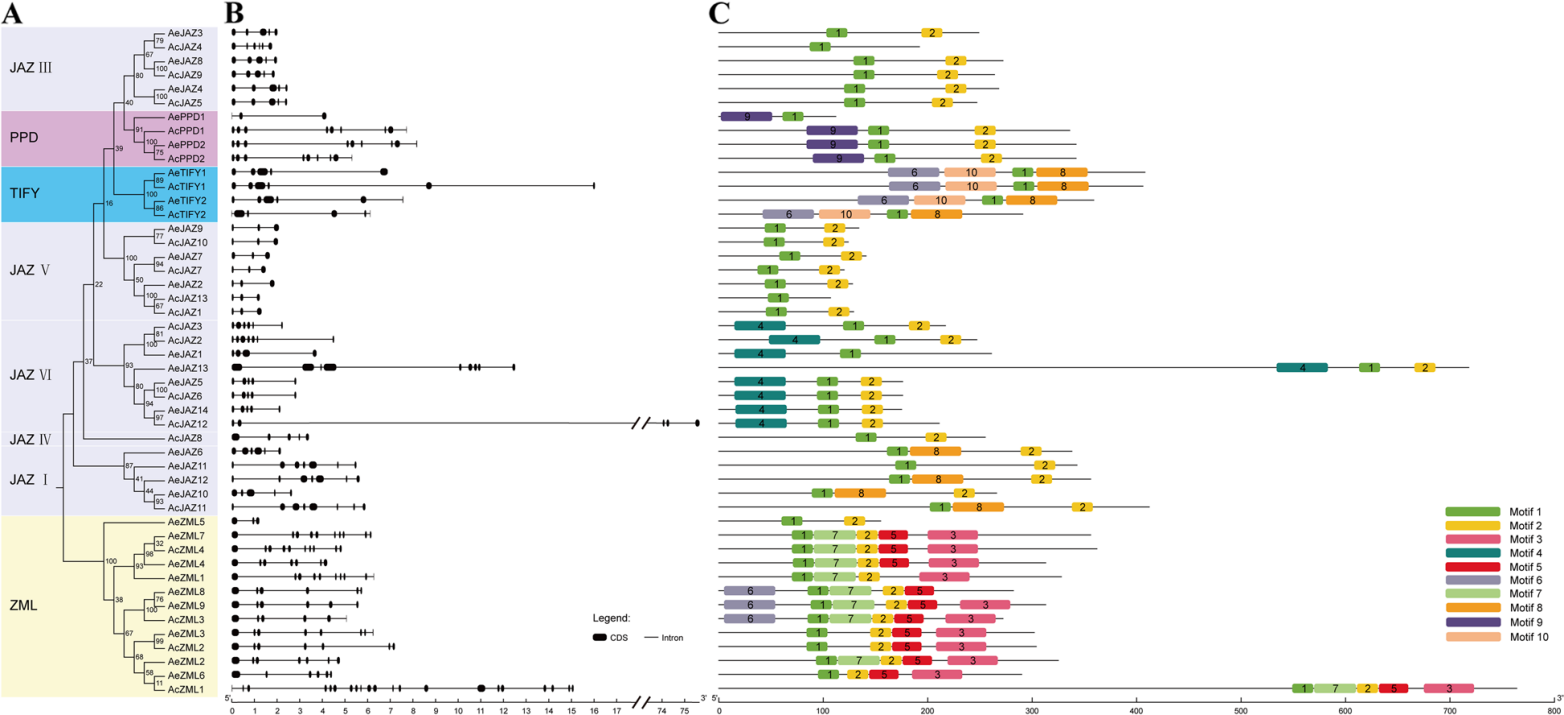
Phylogenetic tree, gene structure and conserved motif of kiwifruit *TIFY* gene family. **A** The neighbor-joining phylogenetic tree of kiwifruit *TIFY* family. **B** Exon-intron structure of kiwifruit *TIFY* genes. Black boxes indicate exons, and black lines indicate introns. The scale bar indicates 1 kb. **C** Distribution of the conserved motifs in kiwifruit TIFY proteins. Ten conserved motifs are marked with different colored boxes. The scale bar indicates 100 aa

To figure out the classification and evolutionary relationships of the identified kiwifruit *TIFY* gene family, a phylogenetic tree was constructed based on the multiple sequence alignment of 86 TIFY protein sequences, including 27 TIFYs in *A. eriantha,* 21 in *A. chinensis*, 18 in *Arabidopsis thaliana* and 20 in rice (Fig. 2). According to the constructed phylogenetic relationship, the 86 TIFYs were classified into four major phylogenetic groups: JAZ, ZML, TIFY and PPD. Among which the JAZ was the largest group with 54 TIFY family members, and could be further clustered into six subgroups (JAZ I to JAZ VI) (Fig. 2). Each of the six JAZ subgroups contained different numbers of JAZ proteins from *A. eriantha, A. chinensis*, *Arabidopsis* and rice. For example, the JAZ I, JAZ III, JAZ V and JAZ VI subgroups all contained members of JAZ proteins from the four species. However, the JAZ II subgroup only contained seven JAZ proteins from rice, and no JAZ protein from other species was clustered in this subgroup (Fig. 2). In addition, no JAZ protein from *A. eriantha* was found in the subgroup of JAZ IV. The group ZML was the second largest group and contained three ZML proteins from *Arabidopsis*, four ZML proteins from rice, nine ZML proteins from *A. eriantha*, four ZML proteins from *A. chinensis*. The TIFY subgroup consisted of six TIFY members, including two TIFY proteins from each of *A. eriantha* and *A. chinensis*, and one TIFY member from each of *Arabidopsis* and rice. The PPD subgroup contained two *A. eriantha* PPD proteins, two *A. chinensis* PPD proteins and two *Arabidopsis* PPD proteins, but did not contain rice PPD proteins (Fig. 2). In addition, phylogenetic analysis was also performed using only the alignment of the 48 kiwifruit TIFY protein sequences characterized herein (Fig. 3A). The topology of the resulting phylogenetic tree indicated that TIFY proteins from the same group tended to be clustered together, which was similar to that of the above phylogenetic tree constructed by TIFY sequences from the four plants (Fig. 2, Fig. 3A).

### Sequence analysis of kiwifruit *TIFY* family

The exon-intron structure of all the identified kiwifruit *TIFY* genes was investigated to better understand the structural diversity of these genes. As shown in Fig. 3B, there were some differences in the number of exons and the length of introns among members of the *TIFY* gene family. For example, the number of exons varied from 3 to 21, and the *AcZML1* had 21 exons, which was much more than that of other *TIFY* genes. Furthermore, the second intron of *AcJAZ12* was the longest among all the sequences, which resulted in the length of the genomic DNA sequence reaching 75.656 kb. The gene exon-intron organization also showed that the structural differences of genes in the same group/subgroup were small, and they often have similar gene structures and exon/intron numbers (Fig. 3B). For example, the genes in the subgroup JAZ IV and JAZ V had five and three exons, respectively. In the JAZ III subgroup, only *AcJAZ4* had six exons, while the other nine genes in this subgroup had five exons. In the PPD group, except *AePPD1* which only contained three exons, all other genes contained nine exons. In the TIFY group, the number of exons varied from four to six, and most genes had six exons (Fig. 3B).

To further reveal the structural and functional characteristics of kiwifruit TIFYs, the online website MEME was employed to analyze the conserved structural motifs of the TIFY proteins. A total of 10 conserved motifs were identified, and named motif one to motif 10. As showed in Fig. 3C, no member of the kiwifruit TIFY family had a complete set of 10 conserved motifs, the motif number of the TIFY family members ranged from one to six. Members of the ZML group generally contained more motifs, and eight out of 13 ZML members contained more than five motifs, while members of other groups or subgroups contained less than four motifs. Of the 10 conserved motifs, only motif one existed in all kiwifruit TIFY members, while motif two also existed in most members (Fig. 3C). In addition, some motifs existed only in specific groups, such as motif four only existed in JAZ VI subgroup, motif nine only existed in PPD group, motif 10 only existed in TIFY group. In general, members of the same group or subgroup usually had the same type and number of motifs, and the distribution of the motifs was often the same. For example, the members of TIFY group mainly contained four motifs, the members of PPD group contained three motifs, members of JAZ I subgroup mainly contained three motifs, and most members of JAZ III subgroup mainly contained two motifs (Fig. 3C).

**Fig. 4:**
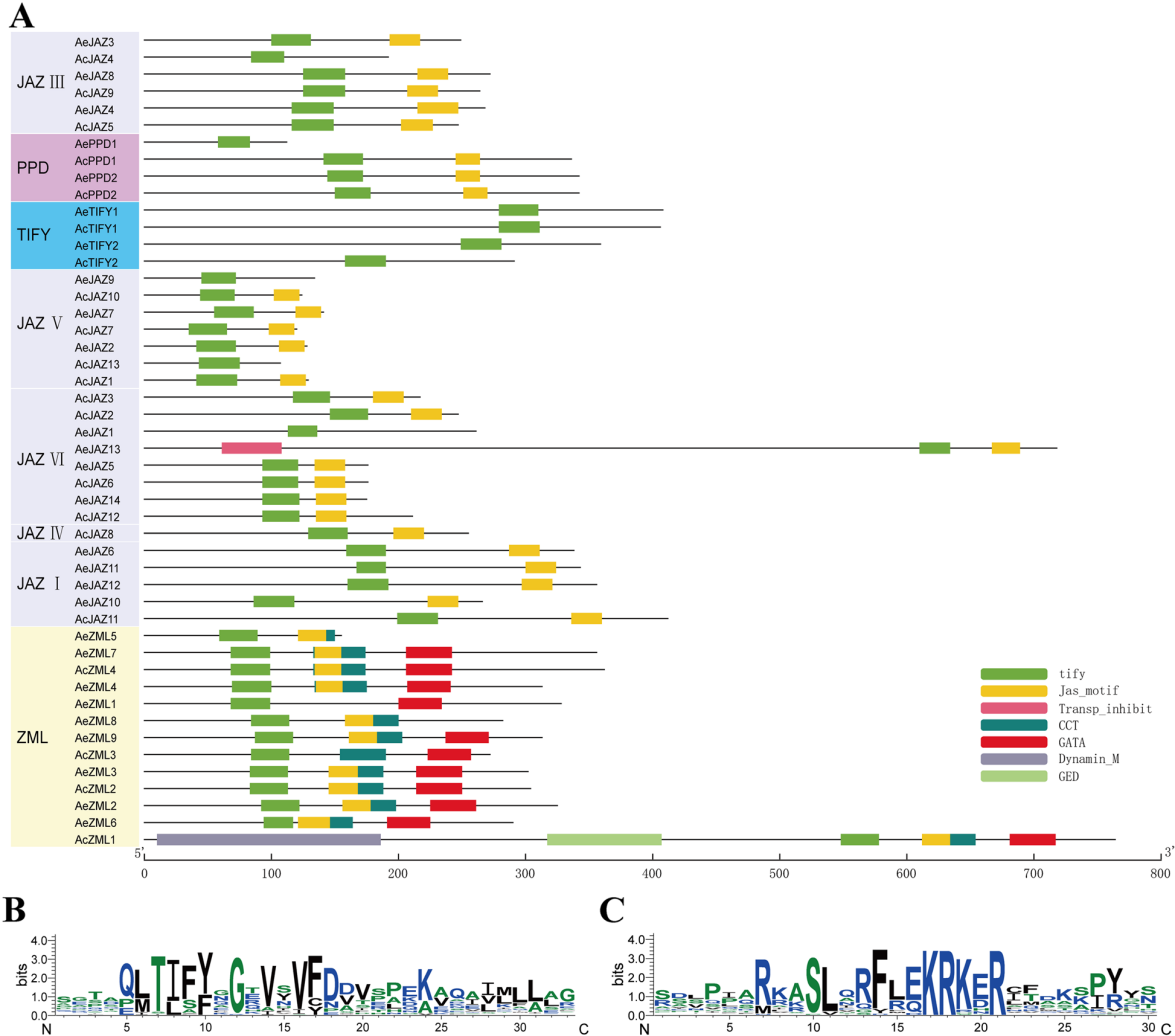
Conserved domain distributions and sequences logos of kiwifruit TIFY family. **A** The distribution of conserved domains in kiwifruit TIFY proteins. Different conserved domains are indicated by different colored boxes. The scale bar indicates 100 aa. **B** Sequence logo of the TIFY domain. **C** Sequence log of the Jas domain

In addition, the conserved domains in kiwifruit TIFYs were also examined using the Pfam web server, and seven putative conserved domains were identified, namely the TIFY domain, CCT domain, GATA domain, Jas_motif domain (also named CCT_2), Dynamin_M domain, GED domain and Transp_inhibit domain as showed in Fig. 4A. Among them, the Dynamin_M and GED domains only existed in AcZML1, and Transp_inhibit domain only existed in AeJAZ13. These three domains may be sequence specific functional domains. Other conserved domains of TIFY, CCT, GATA and Jas, however, tended to exist in multiple sequences and were group/subgroup specific. For example, most members of the ZML group contained four conservative domains, including TIFY, CCT, GATA and Jas. The JAZ group (including five subgroups) mainly contained TIFY and Jas domains, and the PPD group also mainly contained the two domains of TIFY and Jas, while the TIFY group only contained TIFY domain (Fig. 4A). The conserved TIFY domain was found in all the TIFY family members, and the Jas domain was also found in most TIFY members (Fig. 4A).

To further display the conservation patterns of the conserved domains of TIFY and Jas, their sequence logos were generated as showed in Fig. 4B and C. The TIFY domain logo revealed that the TIFY domains were not well conserved, but most of them shared common motifs, such as TIF[Y/F]XG, TLXFXG, SLSFQG (Fig. 4B). Among these conserved motifs, the motif TIF[Y/F]XG was shared by the group members of PPD, TIFY and JAZ, while the motifs of TLXFXG and SLSFQG were only shared by the ZML members (Additional file 1). The TIF[Y/F]XG was the most dominant motif, and 33 out of 48 TIFY family members contained this motif (Additional file 1). Compared with TIFY domain, the Jas domain were more conserved, and shared more conserved residues at the motif of SLX2FX2KRX2R (Fig. 4C).

### Analysis of the promoter cis-elements in the *TIFY* gene family

In order to explore the possible expression regulation patterns of the kiwifruit *TIFY* genes, the cis-elements in the promoter regions of the *TIFY* family members were predicted. The results showed that various putative cis-elements that are involved in stress-response, phytohormone, and plant growth and development were identified widely in the promoter sequence of each kiwifruit *TIFY* gene (Additional file 2). The promoter region of the *TIFY* gene family mainly contained six types of stress-response related cis-elements, including MYB binding site involved in drought-inducibility (MBS), low-temperature responsiveness element (LTR), anaerobic induction element (ARE), defense and responsiveness element (TC-rich repeats), wound-responsive element (WUN-motif) and light responsiveness element (G-box). Among them, the cis-elements ARE and G-box were the most distributed in the kiwifruit *TIFY* families. In the *AeTIFY* family, 26 members each contained these two cis-elements, and in the *AcTIFY* family, 19 members each contained these two cis-elements. The phytohormone related cis-acting elements primarily involved in the gibberellin-responsiveness (P-box, GARE-motif, TATC-box), MeJA-responsiveness (CGTCA-motif, TGACG-motif), auxin responsiveness (AuxRR-core, TGA-element), abscisic acid responsiveness (ABRE), salicylic acid responsiveness (TCA-element). Although three types of gibberellin-related cis-elements were identified in the TIFY promoter regions of kiwifruit, the number of *TIFY* gene family members containing gibberellin related cis-elements was less than that of MeJA, ABA and salicylic acid related cis-elements. As showed in Additional file 2, there were 17, 21, 23 and 16 members in the *AeTIFY* family, and 17, 17, 20 and 10 members in the *AcTIFY* family, containing MeJA (CGTCA motif), MeJA (TGACG motif), ABA and salicylic acid related cis-elements, respectively. In addition, the *TIFY* gene promoters also contained some cis-acting elements related to plant growth and development, including zein metabolism regulation (O2-site), meristem expression (CAT-box), meristem specific activation (CCGTCC-box), circadian control (circadian), endosperm expression (GCN4-motif) and seed-specific regulation (RY-element). Different numbers of regulatory elements were identified in the promoter regions of the *TIFY* genes, such as six to 14 regulatory elements were found in the promoter region of the *AeTIFY* members, and seven to 12 were found in *AcTIFY* members (Additional file 2).

### Chromosomal distribution and gene duplication analyses of the *TIFY* genes

**Fig. 5:**
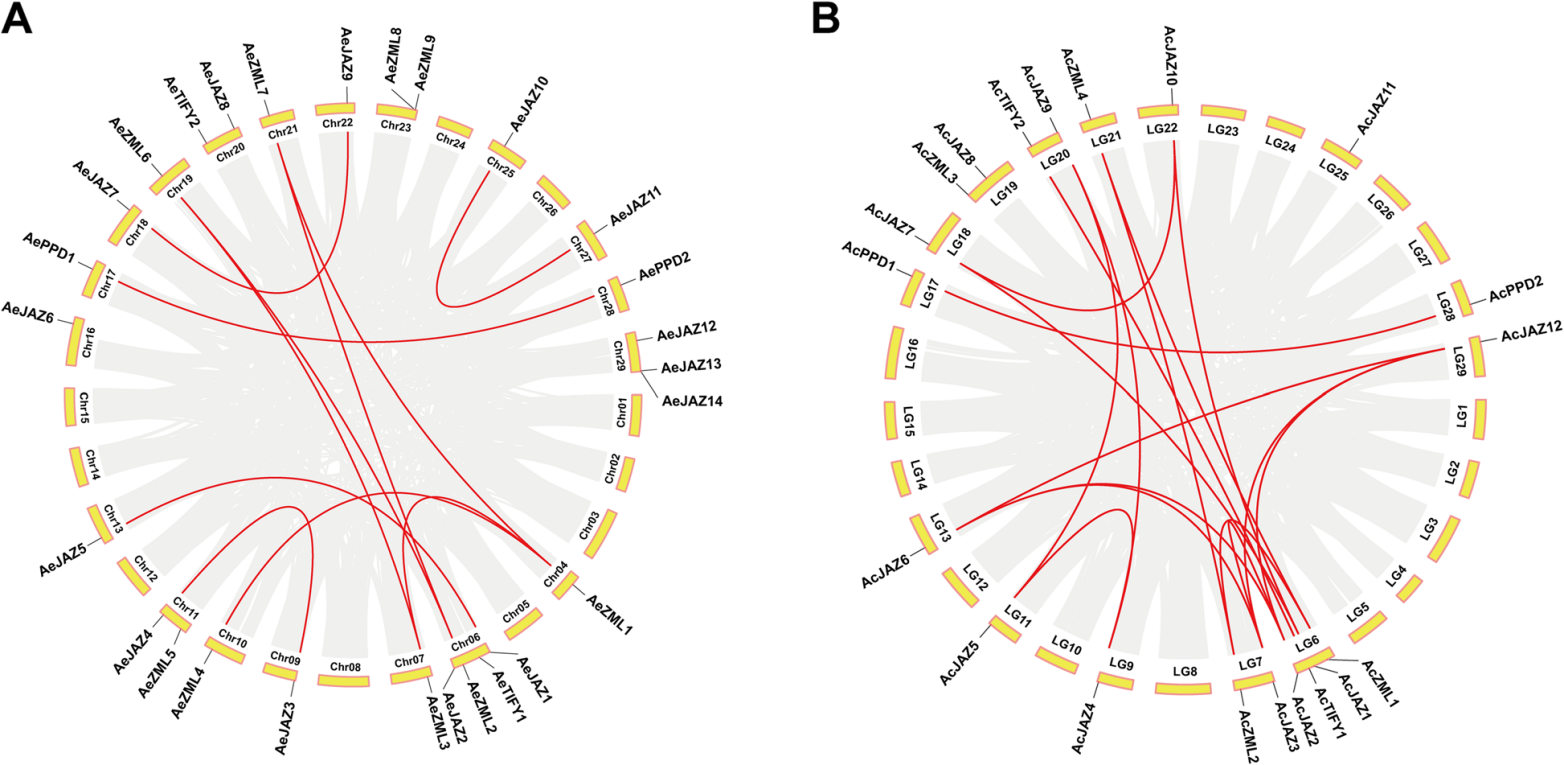
Chromosomal distribution and interchromosomal relationships of kiwifruit *TIFY* genes. **A** Chromosomes of *A. eriantha*; **B** Chromosomes of *A. chinensis* (Hongyang) v3.0. Gray lines indicate all synteny blocks in kiwifruit genomes, and the red lines indicate duplicated *TIFY* gene pairs

The results of chromosomal distribution analyses showed that the *TIFY* genes were distributed irregularly on the kiwifruit chromosomes (Fig. 5). The 27 genes of the *AeTIFY* family members were unevenly distributed on 19 of the 29 chromosomes of *A. eriantha* (Fig. 5 A). Among them, Chr06 had the most genes distributed with four *AeTIFY* members, followed by Chr29 with three *AeTIFY* members. Chr11, Chr20 and Chr23 with two *AeTIFY* members each, and only one *AeTIFY* member on each of the remaining 14 chromosomes (Fig. 5A). Of the 21 *TIFY* genes identified from the *A. chinensis* genome, 20 genes were unevenly distributed on 14 out of the 29 linkage groups (LGs) of *A. chinensis*, and one gene (*AcJAZ13*) was situated on Contig00986 (Additional file 1, Fig. 5B). Among the 14 LGs with *TIFY* gene distribution, LG6 contained four *TIFYs* and had the largest number of *TIFY* genes. LG7, LG17 and LG20 each had two *TIFYs*, and the remaining ten LGs (LG9, LG11, LG13, LG17, LG18, LG21, LG22, LG25, LG28 and LG29) each had one *TIFY* gene.

Several gene duplication events were detected throughout the *TIFY* genes in kiwifruit. The gene pair of *AeZML8*/*AeZML9* in *AeTIFY* family was detected as tandem duplication genes according to previous descriptions of tandem duplication event [40, 41]. However, no tandem duplication event was detected in *AcTIFY* family (Fig. 5, Additional file 3). In addition to the tandem duplication event, 11 and 17 segmental duplication events were also identified with MCScanx method in *AeTIFY* family and *AcTIFY*-v3 family, respectively (Fig. 5, Additional file 3). Among the identified gene duplication events, most of them were segmental duplication events.

The Ka/Ks ratios were calculated to investigate potential selective pressure of the identified duplication gene pairs. The results showed that all the Ka/Ks values of the above detected tandemly and segmentally duplicated *TIFY* gene pairs were less than one (Additional file 3), suggestin**g** that the repetitive *TIFY* genes in kiwifruit were primarily constrained by intense purification selection pressure.

### Synteny and evolutionary analyses of kiwifruit *TIFY* genes and other plants *TIFYs*

To explore the potential evolutionary clues of the kiwifruit *TIFY* gene family, a serious of comparative syntenic graphs of kiwifruit associated with other five representative plant species were constructed. The five representative plant species contained four dicots plants (*Arabidopsis thaliana*, *Camellia sinensis*, *Solanum lycopersicum* and *Vitis vinifera*) and a monocots plant (*Oryza sativa*).

**Fig. 6:**
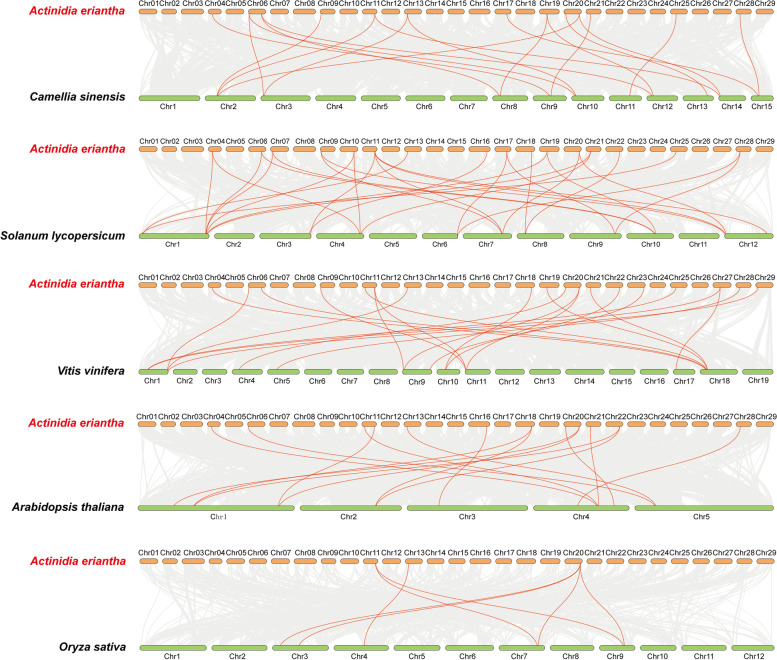
Synteny analysis of *TIFY* genes between *A. eriantha* and five representative plant species. Gray lines in the background indicate the collinear blocks within *A. eriantha* and other plant genomes, while the red lines highlight the syntenic *TIFY* gene pairs

According to the comparative syntenic maps of *A. eriantha* associated with the five plant species (Fig. 6), a total of 19 *AeTIFY* genes showed syntenic relationships with those in *Vitis vinifera,* followed by *Solanum lycopersicum* (16), *Camellia sinensis* (13), *Arabidopsis thaliana* (12) and *Oryza sativa* (3)*.* The numbers of *AeTIFYs* orthologous genes in *Vitis vinifera*, *Solanum lycopersicum*, *Camellia sinensis*, *Arabidopsis thaliana* and *Oryza sativa* were 25, 29, 19, 15 and 7, respectively (Additional file 4). Among the orthologous gene pairs, two *AeTIFY* genes of DTZ79_11g10370 in the syntenic analysis *A.eriantha* and *Solanum lycopersicum* and DTZ79_20g14030 in the syntenic analysis of *A.eriantha* and *Oryza sativa* were identified to be associated with at least three syntenic gene pairs. Although more syntenic gene pairs were identified between *A. eriantha* and dicots than those between *A. eriantha* and *O. sativa*, three collinear gene pairs (DTZ79_11g10370, DTZ79_13g04380, DTZ79_20g14030) were found both in dicots and monocots plants. However, some collinear gene pairs (DTZ79_04g05010, DTZ79_21g05530, DTZ79_28g03670) were just available in dicots plants, but not identified in the monocot plant of *Oryza sativa* (Additional file 4).

**Fig. 7:**
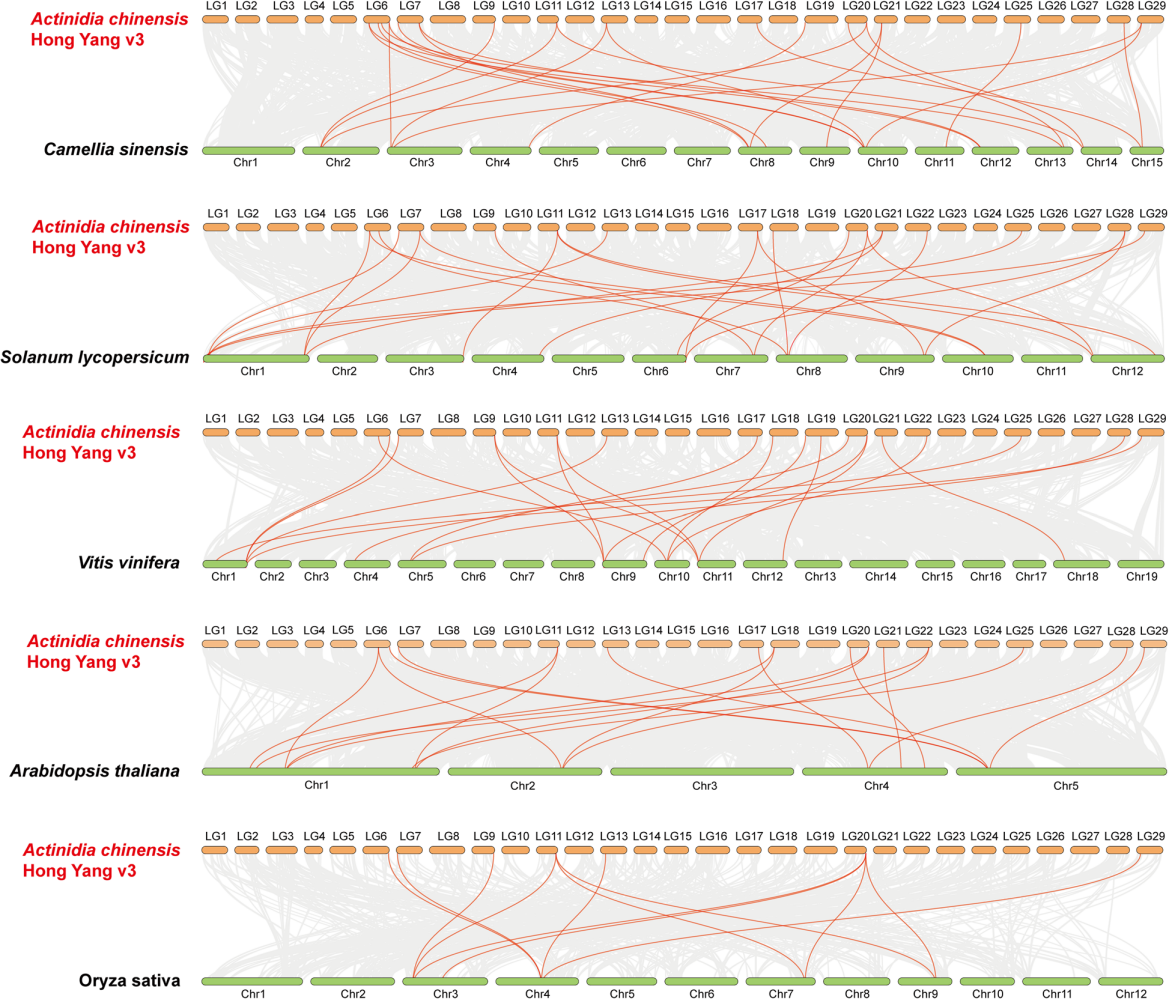
Synteny analysis of *TIFY* genes between *A. chinensis* and five representative plant species. Gray lines in the background indicate the collinear blocks within *A. chinensis* (Hongyang) v3.0 and other plant genomes, while the red lines highlight the syntenic *TIFY* gene pairs

As showed in the comparative syntenic maps of *A. chinensis* (Fig. 7), totally 19 *AcTIFY* family members showed a syntenic relationship with *Vitis vinifera,* followed by *Camellia sinensis* (17)*, Solanum lycopersicum* (16)*, Arabidopsis thaliana* (14) *and Oryza sativa* (7)*.* The numbers of *AcTIFY* genes in *Vitis vinifera, Camellia sinensis, Solanum lycopersicum, Arabidopsis thaliana and Oryza sativa* were 24, 25, 24, 19 and 12, respectively (Additional file 5). In the identified orthologous gene pairs, three *AcTIFY* genes (Actinidia32203.t1 in *A. chinensis* and *Solanum lycopersicum*, Actinidia30242.t1 and Actinidia32203.t1 in *A. chinensis* and *Oryza sativa*) were also found to be associated with at least three corresponding gene pairs. For the syntenic analysis of the *TIFY* genes of *AcTIFY* and the five representative plants, five syntenic gene pairs (Actinidia09609.t1, Actinidia22128.t1, Actinidia30242.t1, Actinidia30601.t1, Actinidia32203.t1) were found both in dicots and monocots plants. However, another six syntenic gene pairs (Actinidia09108.t1, Actinidia10272.t1, Actinidia12384.t1, Actinidia13280.t1, Actinidia18522.t1 and Actinidia19959.t1) were only found in the four dicots plants, but not available in the monocot plant of *O. sativa* (Additional file 5).

**Fig. 8:**
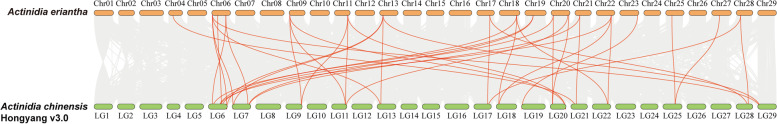
Synteny analysis of *TIFY* genes between different kiwifruit taxa. Gray lines in the background represent the collinear blocks between *A. eriantha* and *A. chinensis* (Hongyang) v3.0 genomes, while the red lines highlight the syntenic *TIFY* gene pairs

In addition, to further display the evolutionary relationships of the kiwifruit *TIFY* gene family, a multicollinearity plot of the *TIFY* genes among the two genomes of *A. eriantha* and *A. chinensis* was drawn (Fig. 8). The results showed that a total of 18 *AeTIFY* genes and 19 *AcTIFY* genes had a collinearity relationship, and 39 collinear gene pairs were identified between *AeTIFY* gene family and *AcTIFY* gene family (Fig. 8, Additional file 6).

Furthermore, the Ka/Ks ratios of all the above identified collinear gene pairs were also calculated, and the results showed that all collinear gene pairs displayed Ka/Ks < 1, indicating that the evolution of kiwifruit *TIFY* gene family might have suffered strong purifying selective pressure (Additional files 4, 5, 6).

### Expression pattern analyses of kiwifruit *TIFY* genes

**Fig. 9:**
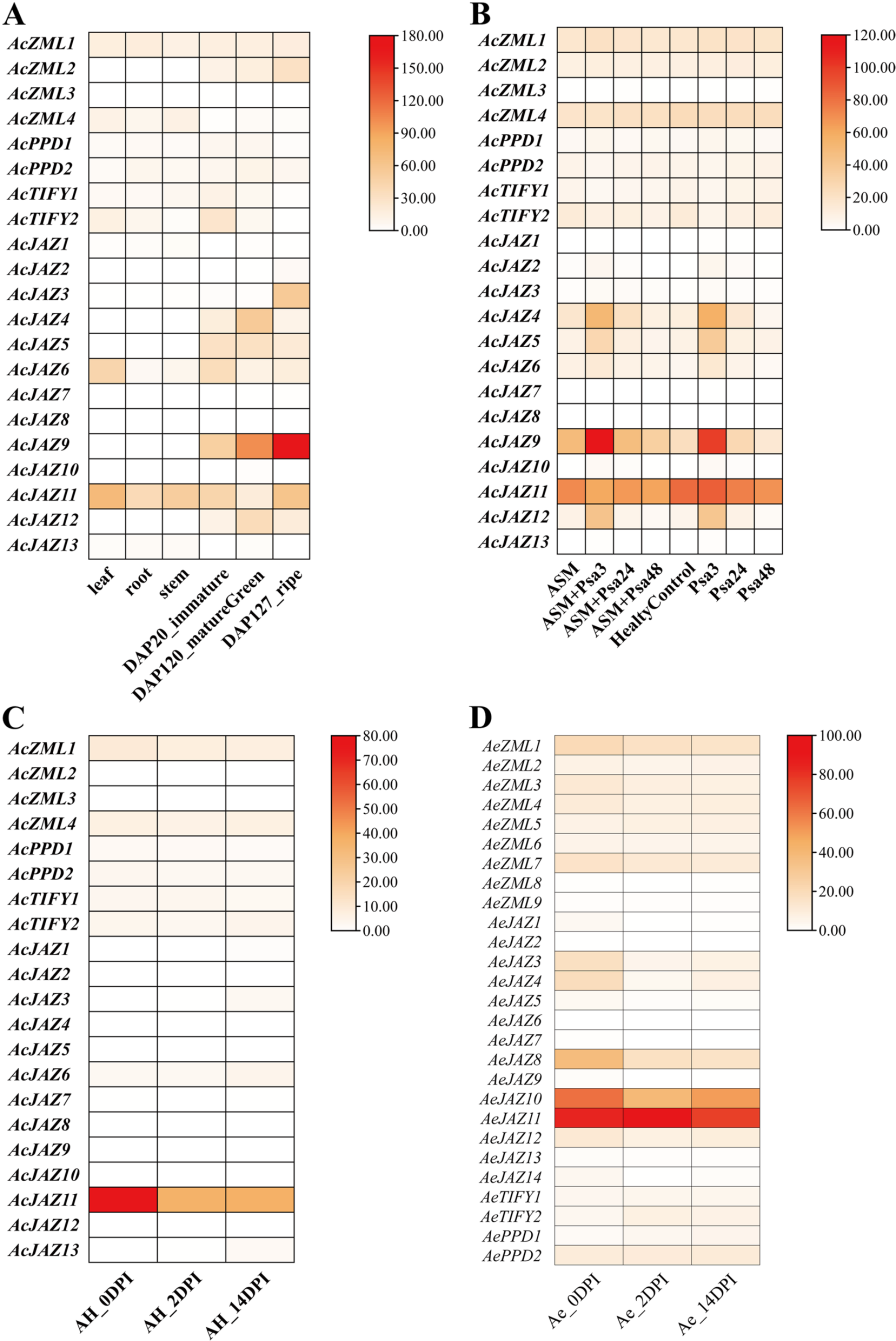
Expression profiles of kiwifruit *TIFY* genes in different tissues, different stages of fruit development and Psa invasion. **A** Expression profiles of *AcTIFY* genes in different tissues and different stages of fruit development. Leaf, root and stem indicate different tissues in *A. chinensis*. DAP20_immature, DAP120_mature green and DAP127_ripe represent different stages of fruit development after pollination. **B** Expression profiles of *AcTIFY* genes in response to Psa infection and ASM (acibenzolar-S-methy) treatment. HealtyControl represents samples without Psa inoculation or ASM treatment. Psa3, Psa24 and Psa48 represent hours post inoculation of Psa. **C** Expression profiles of *AcTIFY* genes in the process of Psa invasion. AH_0DPI, AH_2DPI, AH_14DPI represent days post inoculation with Psa in *A. chinensis*. **D** Expression profiles of *AeTIFY* genes in the process of Psa invasion. Ae_0DPI, Ae_2DPI, Ae_14DPI indicate days post inoculation with Psa in *A. eriantha.* The expression values of kiwifruit *TIFYs* were normalized to FPKM (fragments per kilobase of exon per million mapped fragments)

In this study, the expression patterns of kiwifruit *TIFY* genes in different tissue parts, different fruit development stages and Psa invasion stages were investigated. The expression patterns of the *A. chinensis TIFY* family genes in three main tissues (root, stem and leaf) were invested based on previous RNA-seq data. As showed in Fig. 9A, most of the *TIFY* genes were expressed at different levels in these three tissues, and the expression of some genes is highly tissue-specific. Interestingly, compared with other family members, *AcZML1*, *AcZML4*, *AcTIFY2*, *AcJAZ11* and *AcJAZ6* had relatively higher expression levels. Among them, *AcJAZ11* had a high expression level in root, stem and leaf, and the expression level in leaf and stem was particularly prominent, indicating that this gene may be necessary for the development of tissues such as leaf and stem. *AcJAZ6* was specifically highly expressed in leaf, suggesting that this gene may play an important role in leaf. Similar to *AcJAZ6*, the expression level of *AcTIFY2* in leaf was also higher than that in root and stem. *AcZML1* and *AcZML4* were expressed in all expressed in these three tissues, and no obvious tissue-specific expression pattern was observed (Fig. 9A).

The expression patterns of *A. chinensis TIFY* gene members during fruit development and ripening (DAP20_immature, DAP120_mature green and DAP127_ripe) were further analyzed according to previous transcriptome data. As the results showed in Fig. 9A, the expression levels of *AcZML2* and *AcJAZ9* increased gradually along with fruit ripening, and the change trend of *AcJAZ9* gene expression was more obvious. However, the expression levels of *AcTIFY1* and *AcTIFY2* decreased gradually with fruit ripening (Fig. 9A). In addition, there were also some genes that had higher expression levels at specific stages of fruit development. For example, the expression of *AcJAZ11* was higher in immature and ripe stages, but lower in mature green stage. *AcJAZ3* was highly expressed in ripe stage, while *AcJAZ9* was highly expressed in mature green stage and ripe stage (Fig. 9A). The diversity of *AcTIFYs* expression patterns during fruit development indicated that these genes may played different roles along with fruit ripening.

The expression patterns of *TIFY* family members in *A. chinensis* and *A. eriantha* after the invasion of Psa were detected. The expression profile of *AcTIFYs* with or without ASM treatment in the process of Psa infection was shown in Fig. 9B. The expression levels of *AcTIFY* genes changed in varying degrees after Psa invasion. *AcJAZ12*, *AcJAZ9*, *AcJAZ4* and *AcJAZ5* had similar expression patterns. These genes all had high expression levels in the early stage of Psa infection, and the expression of these genes gradually decreased with the increase of infection. *AcJAZ11* maintained a high expression level during the whole process of Psa infection. The expression of *AcJAZ11* was decreased after ASM treatment compared with the samples without ASM treatment. On the contrary, the expression of *AcJAZ9* after ASM treatment was higher than that without ASM treatment (Fig. 9B). Figure 9C was also the expression profile of *AcTIFY* genes to the invasion of Psa. Similar to the expression pattern of *AcJAZ11* in Fig. 9B, *AcJAZ11* in Fig. 9C also had the highest expression level in the early stage of Psa infection (AH_0DPI), and the expression level of this gene gradually decreased with the increase of infection time (Fig. 9C). Figure 9D was the expression profile of *AeTIFY* genes to the invasion of Psa. The expression levels of *AeJAZ10* and *AeJAZ11* genes remained at a high level at different periods after infection, while the expression level of *AeJAZ11* was the highest in different periods (Fig. 9C), which also suggested *AeJAZ11* might play a certain role in Psa infection.

## Discussion

The *TIFY* gene family, as unique transcription factor family in plants, plays pivotal roles in regulating plant development, physiological processes and response to stresses. In the current study, the *TIFY* gene families in kiwifruit, a fruit crop with important economic values and popular worldwide, were systematically identified and analyzed.

The *TIFY* genes have been identified and characterized in several important horticultural crops, such as grape [28], apple [29], tomato [25, 30], pear [31], watermelon [32], tea [33]. The genus *Actinidia* contains 54 species and 74 taxa, but only a few species with great utilization values have been domesticated, such as *A. chinensis*, *A. deliciosa*, *A. arguta* and *A. eriantha*. At present, the commercial species widely cultivated in the world are *A. chinensis* and *A. deliciosa*, and *A. eriantha* has only a small amount of artificial cultivation. *A. eriantha* is easy to peel, enrich in vitamin C, and has strong resistance to Psa [39]. In this study, the genome-wide identification of the *TIFY* genes of *A. chinensis* and *A. eriantha* were performed. The results showed that there were at least 21 *TIFY* genes in the genome of *A. chinensis*, and at least 27 *TIFY* genes in *A. eriantha* (Fig. 1). The difference in the number of *TIFY* family genes between the two kiwifruit species was mainly due to the difference in the number of JAZ and TIFY group members. The JAZ and ZML groups in *A. chinensis* contained 13 and 4 members, respectively, while JAZ and ZML groups in *A. eriantha* contained 14 and 9 members, respectively (Fig. 1). The difference in the number of gene family members may be due to gene duplication or loss in the process of gene evolution. Gene duplication and loss were the main evolutionary driving forces for the expansion or contraction, and duplicated genes could lead to gene redundancy [42]. Duplication events in the critical sites such as the CDS, and the promoter sequence cause members of a gene family to receive new functions [43, 44].

The *TIFY* genes in kiwifruit genomes showed a high variation in their sequence structure. In terms of protein length, the variation range of amino acid sequence of AcTIFYs was 107 aa to 764 aa, while that of AeTIFYs was 112 aa to 718 aa (Additional file 1). In terms of gene structure, the variation range of kiwifruit *TIFY* gene exon was three to 21 (Fig. 3B). The variation range of the conserved motif of kiwifruit TIFY gene was one to six, and the variation range of the conserved domain of kiwifruit TIFY was also one to six. Some conserved motifs or domains were unique to specific sequences or subgroups (Figs. 3 C and 4A). Those high variation in the sequence structure revealed that *TIFY* family members have acquired changes in their genome during evolution events that affected their functions [43, 45].

Segmental and tandem gene duplications are the two major factors in the generation and maintenance of gene family, and the relative importance of segmental and tandem duplication in the evolution of gene family may correspond to functional differences of the gene family members [46]. In this study, only two tandem duplication events were detected in the kiwifruit *TIFY* gene families, while the rest were segmental duplications. This typical type of low tandem and high segmental duplications is consistent with the classification of gene duplication types in previous works, which mainly includes proteins involved in the roles of transcription factors, signaling, membrane transport and so on [46]. Previous studies have shown that the functions and expression patterns of segmentally duplicated genes were often similar [47, 48]. In this study, the expression pattern of segmentally duplicated gene pairs was not completely consistent, which was different from previous studies. For the duplicated gene pairs of *AcJAZ9*/*AcJAZ4*, the expression of *AcJAZ9* was much higher than *AcJAZ4* during fruit development. During Psa infection, the expression pattern of *AcJAZ9* was also different from that of *AcJAZ4*. In *A. eriantha*, the expression patterns of the duplicated gene pairs *AeJAZ10*/*AeJAZ11* were different, but the duplicated gene pairs *AeZML1*/*AeZML6*, *AeZML3*/*AeZML4* had similar expression patterns. The different expression patterns between duplicated gene pairs indicated that the gene pairs may perform different functions [43]. In addition, the strong purifying selection signals detected in the duplication gene pairs also indicated the functional importance of kiwifruit *TIFY* genes. These results indicated that gene duplication events, especially segmental duplication, contributed to the evolution and expansion of the *TIFY* gene family in kiwifruit species.

The yield and quality of kiwifruit are easily affected by a variety of biotic and abiotic stresses. Abiotic stresses such as salt, temperature and waterlogging often have adverse effects on plant growth and development. Kiwifruit is a salt sensitive plant, and salt stress seriously affects the normal growth and physiological processes of kiwifruit plants [49]. Temperature influences shoot growth and maturation of fruit on kiwifruit. Under high temperature, the contents of carbohydrate and vitamin C in fruit reduced significantly, and the ripening rate and ethylene biosynthesis also decreased, which finally affected the growth and maturation of fruit [50, 51]. In addition, the biosynthesis and transportation of anthocyanins in red-fleshed kiwifruit were also inhibited by high temperature, which affected the nutrition and commercialization of the fruit [52]. Kiwifruit plants are very sensitive to waterlogging stress, which can lead to fruit yield reduction and even plant death in severe cases [53]. In this study, multiple putative cis-elements, which are mainly involved in stress-response, phytohormone, and plant growth and development, were identified from the promoter region of kiwifruit *TIFY* family genes. The diverse cis-regulatory elements in the promoter region of kiwifruit *TIFY* genes indicated that these genes may be involved in response to a variety of stresses and multiple plant hormones response processes, and played a certain role in the growth and development of kiwifruit.

Kiwifruit trees may encounter a variety of biotic stresses during growth, especially the threats of pests and diseases during the growth process. At present, the main threat to the development of kiwifruit industry is kiwifruit bacterial canker. The bacterial canker disease is a devastating disease incited by Psa, which seriously threatens the production and development of kiwifruit industry worldwide [54]. The disease has the characteristics of rapid transmission and strong pathogenicity, and can lead to the death of kiwifruit trees in a large area [55]. However, there is no effective method to prevent and treat the bacterial canker at present. The resistance of kiwifruit varieties and species to bacterial canker disease is very different. For example, *A. chinensis* ‘Hongyang’ and ‘Hort16A’ are highly susceptible to canker disease, while *A. deliciosa* ‘Jinkui’ and *A. eriantha* are highly resistant to canker disease [55, 56]. Therefore, breeding resistant varieties and exploring resistance genes and biological pathways may be an effective way to control the disease. This study analyzed the expression patterns *TIFY* genes in kiwifruit after Psa invasion. The gene expression of *A. chinensis* ‘Hongyang’, which is susceptible to Psa infection, showed that *AcJAZ11* had a higher expression level during the process of Psa infection, but its expression level decreased with the increase of the infection time. *A. eriantha* has strong resistance to Psa, and the gene expression results showed that *AeJAZ10* and *AeJAZ11* had maintained high levels during the whole process of Psa infection. It was worth noting that the expression level of AeJAZ10 in the initial stage of infection is higher than that in other stages of infection, while the expression level of *AeJAZ11* in the initial stage (Ae_0DPI) is lower than that of the Ae_2DPI stage of infection, indicating that the expression level of *AeJAZ11* has a tendency to increase with the increase of infection time. These results indicated that *AeJAZ11* may be a candidate gene in response to Psa infection and play a certain role in the process of Psa invasion. Previous studies have shown that many *JAZ* genes are key regulatory factors in the jasmonic acid (JA) signal pathway, and participate in response to the infection process of a variety of plant pathogens by mediating JA signal transduction [33, 34, 57–61]. The specific mechanism of the candidate gene Ae*JAZ11* in Psa infection, and whether JA pathway plays a role in the process of Psa infection still needs to be further verified in future studies.

## Conclusions

The kiwifruit *TIFY* family genes were comprehensively and systematically characterized in the present study. A total of 27 and 21 *TIFY* family genes were genome-widely identified in the genomes of *A. eriantha* and *A. chinensis*, respectively. The phylogenetic analysis showed that the identified kiwifruit *TIFY* genes could be classified into four main groups of JAZ, ZML, TIFY and PPD, and members within the same group had similar gene structures and motif compositions. Collinearity analysis suggested that segmental duplication events played a major role in the expansion of kiwifruit *TIFY* family genes. The molecular evolution analysis indicated that the evolution of kiwifruit *TIFY* genes were dominated by purifying selection. Promoter cis-elements analysis, spatio-temporal expression pattern analysis and expression characteristics analysis of Psa invasion showed that a few *TIFY* genes might be involved in the growth and development of kiwifruit and the response to Psa invasion stress. The results presented in this study laid a foundation for further exploring and understanding the biological functions of *TIFY* family genes in kiwifruit.

## Methods

### Identification of *TIFY* family genes in kiwifruit genomes

In order to identify the *TIFY* family members from kiwifruit genomes, the whole-genome and proteome data of *A. chinensis* (Hong Yang) v3.0 and *A. eriantha* (White) were downloaded from the Kiwifruit Genome Database (KGD: http://kiwifruitgenome.org/) [62]. The hidden Markov model (HMM) profile of the conservative functional domain of TIFY (PF06200) was obtained from the Pfam database v34.0 (http://pfam.xfam.org/), and the HMM profile was further used to screen the kiwifruit proteomes using the hmmsearch software in the HMMER package v3.0 to obtain the potential gene family members of *TIFY*. After removing redundant and incomplete sequences, the conserved domain architectures of the acquired sequences were further confirmed by Pfam database and SMART website (http://smart.embl-heidelberg.de/). Sequences without the typical functional domain of TIFY were excluded from the dataset, and the amino acid sequences containing the conservative TIFY domain were regarded as potential members of the kiwifruit *TIFY* family and would be used for subsequent analysis. In addition, the TIFY protein sequences of model plants *Arabidopsis thaliana* and rice (*Oryza sativa*) were downloaded from TAIR (https://www.arabidopsis.org/) and TIGR (http://rice.plantbiology.msu.edu/) database, respectively.

The basic physicochemical properties, such as the molecular weight (MW) and isoelectric point (pI) of each kiwifruit TIFY protein, were predicted using the tool of ProtProm in ExPASy (https://web.expasy.org/protparam/). The subcellular localizations of kiwifruit TIFYs were predicted by the web-server of Cell-PLoc 2.0 (http://www.csbio.sjtu.edu.cn/bioinf/Cell-PLoc-2/).

### Sequence alignment and phylogenetic analysis

The multiple sequence alignment was performed using the program of MAFFT v7.409 [63] with auto strategy and default parameter settings. Then the resultant multiple alignment file was imported into MEGA-X software [64] to construct phylogenetic relationship using the Neighbor-Joining (NJ) algorithm, and the tree topology support was assessed by bootstrap analysis with 1000 replicates. Finally, the constructed phylogenetic tree was annotated and visualized using the online tool of EvolView (https://www.evolgenius.info/evolview/) [65].

### Gene structure analysis and conserved motif discovery

The coding sequences (CDS) and their corresponding genomic sequences of all kiwifruit *TIFY* genes were obtained from the KGD, and then submitted to the online tool of Gene Structure Display Server (GSDS, http://gsds.cbi.pku.edu.cn/) to analyze and visualize the exon-intron organization of these genes. The MEME web server v5.33 (https://meme-suite.org/meme/tools/meme) was used to discover the conserved motif patterns of the identified TIFY proteins in kiwifruit. The maximum number of motifs was set at 10, and the default values were employed for the other parameters. In addition, the conserved domain composition of kiwifruit TIFY proteins were also analyzed using Pfam database and then visualized using TBtools [66]. Furthermore, the sequence logos of the conserved TIFY and Jas functional domains were generated using the web based application of WebLogo (http://weblogo.threeplusone.com/) [67].

### Promoter cis-elements analysis of kiwifruit *TIFY* genes

To explore the putative cis-regulatory elements in the promoter regions of kiwifruit *TIFY* genes, the upstream 2000 bp sequences of the transcription initiation codon of all the *TIFY* genes were extracted from the three kiwifruit genomes, and then the PlantCARE database (http://bioinformatics.psb.ugent.be/webtools/plantcare/html/) was used to predict the cis-elements in the promoter regions of kiwifruit *TIFY* genes.

### Chromosomal locations, gene duplication and synteny analysis

To understand the chromosomal distributions of *TIFY* genes in the three kiwifruit genomes, the chromosomal position information of the *TIFY* genes were obtained from the genome sequence files and the corresponding gene structure annotation information files, which were downloaded from the KGD database. Then, the One Step MCSanX function in TBtools software was adopted to analyze the duplication types and intraspecific collinearity of *TIFY* family members. Finally, the Advanced Circles function in TBtools was used to draw the chromosomal location map and the collinearity relationship of the *TIFY* genes. In addition, the Multiple Synteny Plot function in TBtools was employed to constructed and exhibited the synteny relationship of the orthologous *TIFY* genes obtained from *A. chinensis* and *A. eriantha*. Furthermore, the homology of the *TIFY* genes between kiwifruit and the other five plants (including *Arabidopsis thaliana*, *Camellia sinensis*, *Oryza sativa*, *Solanum lycopersicum* and *Vitis vinifera*) were performed using the Dual Synteny Plot in TBtools. The genome sequences and general feature format files of the five selected plants were downloaded from NCBI (https://www.ncbi.nlm.nih.gov/genome). The Simple Ka/Ks Calculator (NG) of TBtools was used to calculate the nonsynonymous (Ka) and synonymous (Ks) substitution values and the Ka/Ks values of each duplicated *TIFY* gene pairs and the syntenic *TIFY* gene pairs, where Ka/Ks < 1 indicated purifying selection, Ka/Ks = 1 indicated neutral selection, and Ka/Ks > 1 indicated positive selection [68].

### Expression pattern analysis of kiwifruit *TIFY* genes

To explore the expression patterns of kiwifruit *TIFY* genes, the RNA-seq expression profiles of the *TIFY* genes were mined from the KGD database under the project of PRJNA187369, PRJNA328414 and PRJNA436459. The PRJNA187369 project is the transcriptome analysis of *A. chinensis* ‘Hongyang’ leaves and fruits at different developmental stages [36]. The PRJNA328414 project is the transcriptome data of different kiwifruit taxa (*A. chinensis* ‘Hongyang’ and *A.eriantha*) infected by the pathogen Psa of kiwifruit canker disease [69, 70]. The PRJNA436459 project is the transcriptome analysis of *A. chinensis* with or without ASM (acibenzolar-S-methyl) treatment during the inoculation of Psa [71]. The heat maps of the expression levels of the *TIFY* genes were visualized using the Heatmap illustrator program in the toolkit of TBtools.

## Supplementary Information

**Additional file 1. **List of the identified *TIFY* family genes in kiwifruit.

**Additional file 2. **Analysis of cis-elements in promoter regions of kiwifruit *TIFYs*.

**Additional file 3. **Segmentally and tandemly duplicated kiwifruit *TIFY* gene pairs.

**Additional file 4. **One-to-one orthologous relationships between *A. eriantha* and other five plant species.

**Additional file 5. **One-to-one orthologous relationships between *A. chinensis* and other five plant species.

**Additional file 6. **The homologous relationships between *A. eriantha* and *A. chinensis*.

## Data Availability

The kiwifruit genome sequences of *A. chinensis* (Hong Yang) v3.0 and *A. eriantha* (White) were downloaded from the Kiwifruit Genome Database (KGD: http://kiwifruitgenome.org/), and the RNA-seq data were also downloaded from KGD. The TIFY protein sequences of *Arabidopsis thaliana* and rice were downloaded from TAIR (https://www.arabidopsis.org/) and TIGR (http://rice.plantbiology.msu.edu/) databases, respectively. The genome sequences and general feature format files of the five selected plants (*Arabidopsis thaliana*, *Camellia sinensis*, *Oryza sativa*, *Solanum lycopersicum* and *Vitis vinifera*) were downloaded from NCBI (https://www.ncbi.nlm.nih.gov/genome).

## Abbreviations

MW: Molecular weight
pI: Isoelectric point
CDS: Coding sequences
GSDS: Gene Structure Display Server
KGD: Kiwifruit Genome Database
Ka: Nonsynonymous
Ks: Synonymous
DPI: Days post inoculation
Psa: *Pseudomonas syringae* pv. *actinidiae*
JA: Jasmonic acid
ASM: Acibenzolar-S-methyl
DAP: Days after pollination
HMM: Hidden Markov model
